# Use of Blockchain Technology in the Domain of Physical Exercise, Physical Activity, Sport, and Active Ageing: A Systematic Review

**DOI:** 10.3390/ijerph19138129

**Published:** 2022-07-02

**Authors:** Juan Lopez-Barreiro, Luis Alvarez-Sabucedo, Jose Luis Garcia-Soidan, Juan M. Santos-Gago

**Affiliations:** 1 Faculty of Education and Sport Sciences, Campus A Xunqueira, s/n, University of Vigo, 36005 Pontevedra, Spain; jlsoidan@uvigo.es; 2 AtlanTTic, Campus Lagoas-Marcosende, University of Vigo, Maxwell, s/n, 36310 Vigo, Spain; lsabucedo@det.uvigo.es (L.A.-S.); jsgago@det.uvigo.es (J.M.S.-G.)

**Keywords:** health promotion, DLT, training, sport activity, eHealth, fitness

## Abstract

Blockchain technology provides a distributed support for information storage and traceability. Recently, it has been booming in a wide variety of domains: finance, food, energy, and health. In the field of physical activity, physical exercise, sport, and active ageing, this technology could also originate some interesting services introducing support for reliable repository of results, for gamification, or for secure data interchange. This systematic review explores the use of blockchain in this context. The objective is to determine to which extent this technology has fulfilled the potential of blockchain to bring these new added-value services. The authors explored 5 repositories in search of papers describing solutions applied to the above-mentioned frame. 17 papers were selected for full-text analysis, and they displayed diverse applications of blockchain, such as Fitness and healthcare, Sport, and Active ageing. A detailed analysis shows that the solutions found do not leverage all the possibilities of blockchain technology. Most of the solutions analyzed use blockchain for managing, sharing, and controlling access to data and do not exploit the possibilities of Smart Contracts or oracles. Additionally, the advantages of the blockchain model have not been fully exploited to engage users using approaches such as gamification.

## 1. Introduction

A new paradigm in the storage and management of data burst forth in 2008 under the name of Blockchain (BC). In an article signed under the pseudonym of Satoshi Nakamoto, a revolutionary technology to store and manage information in a P2P framework with high-trust levels was introduced [1].

This innovative step forward is making possible a new paradigm in a broad spectrum of domains [2,3,4,5,6] beyond cryptocurrencies, its best-known application. Among these new areas of application, it is worth mentioning some examples such as stock management, financial management, traceability of operations, or the processing of health records. The latter area mentioned is one of the most significant application niches in the domain of eHealth [7]. This work aims to explore to what extent BC may be a game changer in the context of physical activity for health improvement and active ageing, in a broad sense.

From the point of view of the authors, this area can take advantage of this technology as it provides with features such as decentralization, transparency, open source, autonomy, immutability and anonymity [8]. Using these features, it would be possible to implement services that may play a relevant role in this area, such as, the secure storage of sport results without the need of a reliable third party involving (or not) decisions and consequences; supporting gamification techniques, defined as “the use of game design elements in non-game contexts” [9], among peers; triggering alerts in a fully automatic and decentralized manner on data from sports or from biomedical sensors; and others interesting services that may still be waiting to be discovered by the researchers of the domain. Nevertheless, it must be kept in mind that its inherent drawbacks, such as the difficulty of data deletion or the lack of privacy, should also be properly dealt with. Actually, there are several works that show its application in medicine for the management of electronic health records (eHR) [5,10,11] and how it is possible to make significant contributions in this domain bearing the existing constraints.

The arrival of BC can be categorized as a major revolution in the frame of services on the Web. This new model offers the possibility of outsourcing the reliable storage of data and the support for its traceability without the need for trusted third parties [12].

After its introduction, this technology grew in momentum at high speed. Various milestones were fulfilled and support for new advanced features were developed: new networks with support for information storage of a more diverse nature such as Ethereum, the introduction of complete Turing machines in the network itself through the use of Smart Contracts (SC) [13], the provision of support for this technology through the BC as a service (BaaS) model or the inclusion of oracles that allow the gathering of data from the “real world” to be inserted in the “virtual world” for these networks [14,15,16]. These features support a wide variety of services for the final user such as cryptocurrencies, traceability records in the food industry or digital identity management.

“Blockchain is a shared, immutable ledger that facilitates the process of recording transactions and tracking assets in a business network” [17]. This information, due to the nature of the system itself, becomes immutable. To this end, it relies on a P2P structure in which the nodes that form part of the net collaborate with each other to guarantee the inviolability of the data and its high availability, which is not dependent on the failure of any particular server [8,12]. This last aspect, the non-dependence on a particular server and therefore under the management of a third party, allows it to become the right tool when it is better not to rely on any third party. All the agents in the network themselves validate the records and add them to a chain of data blocks (hence the name of the technology) that constitutes the aforementioned ledger of records [18].

Each time an agent involved in the system intends to introduce a new block in this so-called main ledger, it is necessary for all the nodes of the network to reach an agreement on the validity of this new block. This is done by means of a specific protocol that establishes the objective criteria for the acceptance or rejection of a block in the chain. This protocol, called consensus algorithm, can rely on different features or require different conditions to the nodes proposing the inclusion of a new block in the chain. Among the most popular algorithms of this type, there are two that account for most of these systems. The most common one is the so-called Proof of Work (PoW) [19], which requires nodes (or miners in this model) to solve a complex mathematical problem to justify the inclusion of the new block in the network. This would be the model chosen for the famous Bitcoin network. Also, it is quite common to use the Proof of Authority (PoA) [20] as a consensus algorithm. Under this model, the inclusion of new blocks of records are based on the relevance of the node proposing the new block. This second model is much more efficient in terms of energy consumption (as no complex mathematical problem needs to be solved) but, on the other hand, it can be considered “less democratic” as it privileges certain pre-selected nodes whose privileges are different from the other nodes on the net. Also, in addition to these models mentioned, there are a myriad of different algorithms. Among the most prominent, it is worth mentioning Proof of Stake (PoS) [19], Delegated Proof of Stake (DPoS), PoB (Proof of Burn) [21], Practical Byzantine Fault Tolerance (PBFT) [22], Istanbul Byzantine Fault Tolerance (IBFT) [23], or the Raft Consensus [24]. It can be considered that the design of new algorithms for this purpose is an area of active research.

Besides allowing data storage using this distributed information paradigm, BC networks offer other technical features that can provide value-added services. In particular, it is convenient to highlight two remarkable tools in this environment:

- Smart Contracts (SC): This feature provides to these, in principle, “passive” information storage networks with the possibility of proactively making decisions in the face of changes in their environment. By means of these scripts, the network is provided with the possibility of carrying out transactions and making decisions autonomously. In other words, the network nodes themselves carry out the planned operations on the data. This operation does not only fulfill without any human interaction but, as a matter of fact, no human intervention may prevent them from being executed even if they want to [13].
- Oracles: These agents are in charge to capture data from the real world and to introduce it in the ledger of the blockchain platform considered. This way, SCs are able to also use that information for taking the decisions implemented on their code. These agents, whether software or hardware, are considered to be trustable and, usually, require no human intervention for capturing and inserting data [16].

In the frame of this technology and due to the features already mentioned, a new concept was introduced recently. As a way to assign unique or non-fungible items to a certain user, the so-called Non-Fungible Tokens (NFT) were introduced. These units of data stored on the BC prove that a certain user is in possession of a token that is unique, trackable, and interchangeable. Even though they can be considered as a speculative asset in certain contexts, it may be quite useful in some scenarios, for example, to reward users in gamification contexts [25].

This new paradigm also is quite adequate for the delivery of the final solutions using non-conventional approaches. As a matter of fact, in order to fully exploit the potential of this model, it is common to make use of the so-called Decentralized Applications (DApps), either for mobile environments or for conventional ones. These apps may be hosted on a P2P environment using BC (or other P2P network) as the backend for data and for logic, relying on rules codified as SC [26,27].

These enhanced capabilities may make BC preferable to other alternatives that have been known for a long time, such as relational databases or NoSQL models. These technologies, as the reader may note, just provide support for data storage but are deprived of the additional features of BC already described in Section 1. However, it is important to note that these technologies are not mutually exclusive, i.e., in many contexts the combined use of several of these technologies leads to interesting outputs for the project. It may be also noted that in many environments the use of BC technology does not seem fully justified. This is due to the fact that the specific services offered by BC are not fully used and the additional cost derived from the complexity required by this type of solutions is not justified.

Practitioners of the domain have also to face an issue regarding privacy. Due to the nature of the BC, all nodes in the P2P network are aware of the data included in each block of data. If countermeasures are not considered, this may pose an issue regarding ethical and legal constraints. In particular, General Data Protection Regulation, for the European domain (GDPR) [28], Personal Information Protection and Electronic Documents Act., in Canada (PIPEDA) [29], Health Insurance Portability and Accountability Act., in USA (HIPAA) [30], and Personal Information Protection Law, in China (PIPL) [31] present some mandatory features and services that must be considered in any solution presented to be used in their correspondent scopes. This may not be something obvious. As a matter of fact, some authors claim for a reconsideration of these schemes in the light of the potential problems with this technology [32].

Also, a noteworthy obstacle that may prevent its adoption is the low maturity of this technology. The fast rate of obsolescence of tools and protocols versions may be an issue for the maintenance of solutions, given the low number of professionals properly trained in this technology. Nevertheless, presently, cloud-based services for BC are becoming more and more popular [33]. As a matter of fact, in recent times, it is possible to host a BC under the model Blockchain as a Service (BaaS). This way, the setting and managing of the BC net is completely externalized from the project [15].

By focusing efforts within the area of eHealth in a broad way and knowing the health benefits produced by the practice of physical activity, the aim of this systematic review is to explore to what extent and how this technology has been leveraged in relation to physical exercise (PE), physical activity (PA), sport and active ageing (AA).

These terms have been misunderstood over time. Therefore, the following are definitions of each of them. PA is classically defined as any bodily movement produced by skeletal muscles that results in energy expenditure [34]. PE is a subset of PA that is planned, structured, and repetitive and has as a final or an intermediate objective the improvement or maintenance of physical fitness [34]. Sport is any human physical and intellectual activity, competitive in nature, and governed by institutionalized rules [35]. And finally, AA as defined by the World Health Organization (WHO), “Active ageing is the process of optimizing opportunities for health, participation, and security in order to enhance quality of life as people age. Active ageing applies to both individuals and population groups” [36].

The motivation for this study arises from its obvious potential for a world with an increasing average age, material possibilities for preventive and health-oriented sport practice, as well as a high penetration of Information and Communication Technology (ICT). In particular, it is intended to address the problem posed in a generic way, through the research questions shown in Table 1.

To answer these questions, the scientific literature was surveyed to identify the published articles since 1 January 2018 that discuss novel research targeted at the introduction of BC in the field of PE, PA, sport, and AA. This study would ultimately serve to highlight the potential of BC in the already explored or unexplored field of PE, PA, sport, and AA; to detect existing gaps, and to provide the groundwork for new lines of research in this domain.

Section 2 of this document discusses the methodology and tools applied to identify the relevant scientific literature to answer the research questions posed. Section 3 describes the results of applying this methodology, namely the identification of 17 relevant articles fulfilling the requirements established in Section 2. Section 4 shows the analysis of the articles selected. Finally, Section 5 offers the conclusions drawn from this systematic review

## 2. Materials and Methods

This review was driven by the general principles of the Preferred Reporting Items for Systematic Reviews and Meta-Analysis (PRISMA) [37]. According to this approach, a search strategy was defined, eligibility criteria were established, and a selection process was followed. As a result, a corpus of documents was obtained that will allow us to obtain results in terms of the proposed search.

### 2.1. Search Strategy

The following normative databases were used to conduct the search process for the PRISMA start-up phase on 20 October 2021: Web of Science (WoS), ProQuest, Pubmed, Scopus and SportDiscus.

The objective of the search was to locate studies that addressed (1) the use of blockchain technology and (2) applied in the field of PE, PA, sport, and AA.

According to the search requirements, the standard query consisted of two blocks of terms, one for each condition above, linked by logical AND operators. Within each block the terms related to the search condition were linked by logical OR operators:

(“Blockchain” OR “DLT” OR “Hyperledger”) ^(1)^ AND (“endurance training” OR “strength training” OR “resistance training” OR fitness OR growth OR “growing up” OR “healthy life” OR “older people” OR “elder people” OR elderly OR aging OR sport OR exercise OR “physical activity” OR “physical activities” OR “ageing”) ^(2)^

Query results from the databases considered, filtered by title and abstract, were uploaded to Zotero.

An informal search of external sources was also conducted. In particular, papers available on Google Scholar, Slashdot, The Verge, and Reddit were investigated. The results of this process were also incorporated into Zotero.

### 2.2. Eligibility Criteria

Only articles written in English and published from 1 January 2018 to 20 October 2021 that were relevant to answering the research questions were considered.

The following exclusion criteria were used:

1. Works not clearly focused on the field of PE, PA, sport, or AA.
2. Systematic review articles.
3. Doctoral theses.
4. Articles focused on the technical characteristics of the BC protocol itself.

### 2.3. Selection Process

The files corresponding to the searches in each database were imported using the JabRef program to eliminate duplicates. Once these were eliminated, the complete list was exported to a spreadsheet to manage the review of each one of them. The articles were divided into two blocks for their first analysis. In this phase, each of the articles was reviewed by two specialists, one from the technical field and one from the health field.

In the screening phase, based on the information contained in the title and abstract, the relevance of each article to answer the research questions was evaluated, labeling each article on a scale from 0 (not relevant at all) to 3 (totally relevant), as done in another review [38]. Articles with an average of 2.5 or 3 points, made it directly to the next phase. Articles with an average of 0, 0.5 or 1 point, were discarded. Correspondingly, the articles rated with an average of 1.5 or 2 points were evaluated again to decide whether they finally entered the full-text analysis phase or were discarded.

Afterwards, the articles considered for the next phase, full-text analysis, were again assigned to pairs of reviewers from the ICT and health fields, but avoiding the reviewers assigned in the first phase. Again, in case of discrepancies in the scoring, another pair of reviewers would take part again to resolve the decision.

## 3. Results

**Figure 1:**
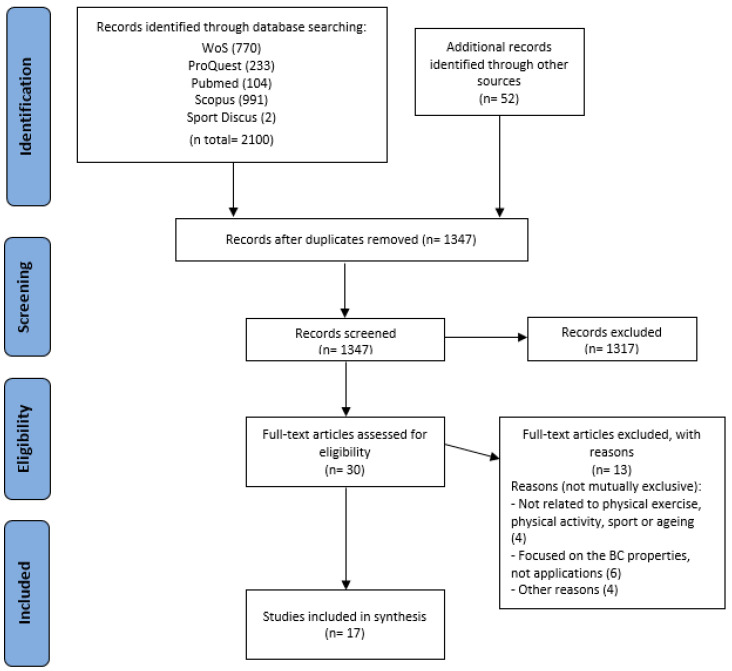
Flow diagram of the systematic review according to PRISMA guidelines.

As above mentioned, the aim of this study was to conduct a systematic review, based on the PRISMA methodology, to analyze and combine the results of selected studies on the novel use of BC technology in the field of PE, PA, sport, and AA. As shown in Figure 1, during the initial search, 2100 articles found in the selected databases and 52 articles found in other sources, 48 of them from Google Scholar using a similar search query, were added. After elimination of duplicates, 1347 articles were screened by title and abstract and rated between 0 and 3 according to their relevance to the research. After rating, in the mean score, 1278 articles received less than 1 point, 40 articles were rated with 2 points, 13 works received 2.5 points and 16 received a 3-point score.

The works rated with an average of 2.5 or 3 points, made it directly to the next phase. Correspondingly, the articles rated with an average of 1.5 or 2 points were evaluated again to decide whether they finally entered the final phase or were eliminated. Finally, 29 papers rated with 2.5 or 3 points, and one paper rated with 1.5 or 2 points, were included in the next phase after being reevaluated by the other pair of reviewers. In total 30 papers were selected for full text reading. Among these 30 papers, 13 were finally eliminated because they did not meet the inclusion criteria. Four papers were eliminated under the label “Not related to PE, PA, sport, or ageing” [32,39,40,41], six under the label “Focused on the BC properties, not applications” [32,42,43,44,45,46], and finally four papers were removed for other reasons (preliminary theoretical proposal, mechanisms for avoidance of fraud in the soccer transfer market, theoretical explanation) [47,48,49,50].

Finally, 17 articles were selected [51,52,53,54,55,56,57,58,59,60,61,62,63,64,65,66,67], and analyzed in depth, focusing mainly on extracting information on: (1) scope; (2) service proposal; (3) Technology readiness level (TRL); (4) information stored; and (5) use of BC properties.

On Table 2 it is shown the definition for each TRL according to the European Union TRL definition [68]. Table 3 summarizes the characteristics of the articles that made it to the final phase. The reader can check the key aspects of each paper and set a starting point for future research in the field of the application of BC technology in the world of PE, PA, sport, and AA. The papers are grouped under three labels according to the field of application “Fitness and healthcare”, “Sport” and “Active ageing”. Under “Fitness and healthcare” we find four works (23.5%), “Sport” includes six papers (35.3%) and “Active ageing” comprises another seven papers (41.2%). TRL is a scale for estimating the maturity of technologies during the acquisition phase of a program based on a scale from 1 to 9, with 9 being the most mature technology. The TRL level assigned to each paper is based on the interpretation of the authors, as it is not explicitly specified in any of them, obtaining a range between level 2 and 6. 

Regarding the contribution of the BC to store data (one the main features of BC), two options were identified: “Actual data”, i.e., information is directly stored on the BC as it is gathered; and “Pointers and hash of actual data”, i.e., information is stored elsewhere, and BC is used to ensure the invariability of information. Fourteen (82.4%) of the selected studies were included in the first case [51,52,53,54,55,56,57,58,59,63,64,65,66,67], and the remaining three (17.7%) fit on the second option [60,61,62]. This decision is highly related to what extent advanced properties of BC are deployed in each particular case. As the reader can note, some works use different properties in line with the actual goal aimed at. The reader can check this in the last column of Table 3 where it can be measured the contribution of the properties applied from BC with the service offered on each work.

In order to provide a more in-depth description of the works included, the following analysis is provided. It specifies the proposal for each paper, the use of BC features and performance analysis if available.

A number of contributions may fit under the label “Fitness and Healthcare”. This way, [51] proposed a platform to boost physical activity and promote a healthy lifestyle through gamification and rewarding users for meeting their goals with cryptocurrencies managed in a BC platform. This could help to promote a more active life by improving the health of users. Regretfully, as the authors mention, malicious users could be able to manipulate the platform to obtain undeserved rewards.

In [53], health data from users is stored in electronic health records (eHR) to make diagnosis and treatment easier and more cost effective. As a limitation of the work, authors report performance issues using the PoW consensus algorithm on Raspberry Pi 3. To enhance the performance, PoA was the final decision as consensus algorithm. Using Jetson TX1, both algorithms work correctly but the energy consumption increases a lot.

Finally, [55,56] assigned training and diet programs to each user based on anthropometric and body composition data. Both used BC to provide security and integrity to sensing data. Furthermore [56] allowed the transfer of user profiles stored in the BC between different sports centers. They reported some issues with throughput and latency when peers and transactions increased. Ref. [55] states that their developed system using permissioned BC network solves some inherent issues, such as data scalability, security, and identity. Also, the SC integrated inference engine used significantly enhances system performance in terms of throughput and resource utilization.

Referring to the category devoted to sport, some works has shown to be relevant for this study. In [67], authors developed a model to improve and guide training using athletes’ physiological data. According to the authors, such a data collection system stored on a BC platform can make manage transactions in an open and transparent manner. Regretfully, as in other works, no assessment on the system performance is included. The model developed by [52] can capture data and predict performance to improve training programs. The work is focused on prediction accuracy but not performance information is included. In [58], the users walking patterns obtained from wearable sensors to design running auxiliary training programs. BC technology was used to design a data transmission and storage plan for the protection and analysis of the user’s personal privacy data. The authors report increases in latency times as security requirements increase. In [59], a model for storing data regarding belt promotions in Taekwondo was developed to keep track of scores and to bring transparency and immutability to that information. Regretfully, they do not mention any performance test or analytic data in this regard. In other similar work, [63], proposed a system for real-time capture and management of athlete fitness data. In this work, BC is used to make data private; regretfully, performance tests are not mentioned. Lastly, [54] captured player performance data to make tactical decisions on the spot and in time. They designed a BC system so, according to the authors, professional sports teams can apply Big Data analysis. Again, no performance analysis is included.

In the frame of active ageing, [65] proposed an application to promote active ageing and assess the level of physical activity practice and quality of life. BC was used to provide privacy, control, and ownership over users’ data; and to support dynamic source reputation and consistent quality of service in complex sensor networks. In [57], it is developed a system that provides alerts if normal patterns of behavior change. They introduced BC to protect data and prevent scams or frauds that might jeopardize people’s privacy. Additionally, [66] proposed a system to persistently control the informed consent in studies powered by BC technology to ensure transparency. None of the above-mentioned papers reported any performance tests.

Following with the authors who researched on active ageing, [61] developed a system to control smart home devices using gesture recognition. They used BC to provide security, trust, digital identity, and privacy preservation. In their work they had latency problems due the high throughput of the gesture tracking algorithm. This data, once captured, is parsed, and sent to the private, permissioned BC, which approves the transaction and add the data to the IPFS storage. The complexity of the model produces an expected larger latency. In [64], physiological data from patients is captured and secured using a BC platform. According to the authors, although there was a small increase in the access time to medical data, it was not significantly impacted by their privacy control based on BC. In other work, [62] determined the risk factors for falls and developed a model to predict them through SCs. By mean of these SCs, they manage the risk values and the priority against each risk factor, then the risk for falls is calculated and alerts sent to interested agents if it is necessary. They used BC to address security and privacy challenges for the data exchange. No report of performance is included. And finally, in [60], a model to assign therapies according to the treatment needs of the users is presented. BC was used to preserve the therapeutic data privacy, ownership, generation, storage, and sharing. Authors conclude that the mean processing delay increases as the number of users increases.

**Figure 2:**
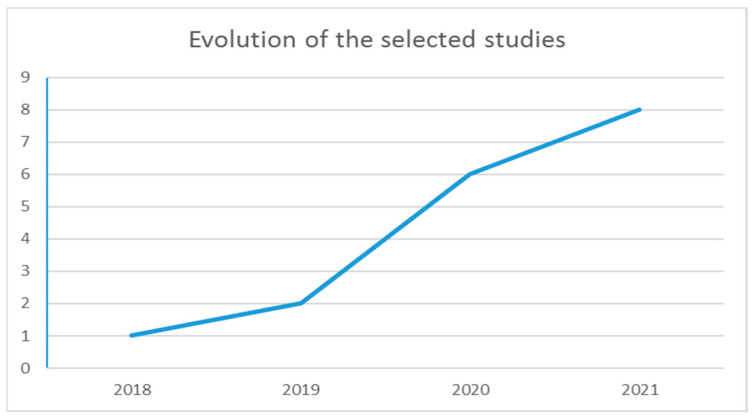
Evolution of the selected studies over the years.

**Figure 3:**
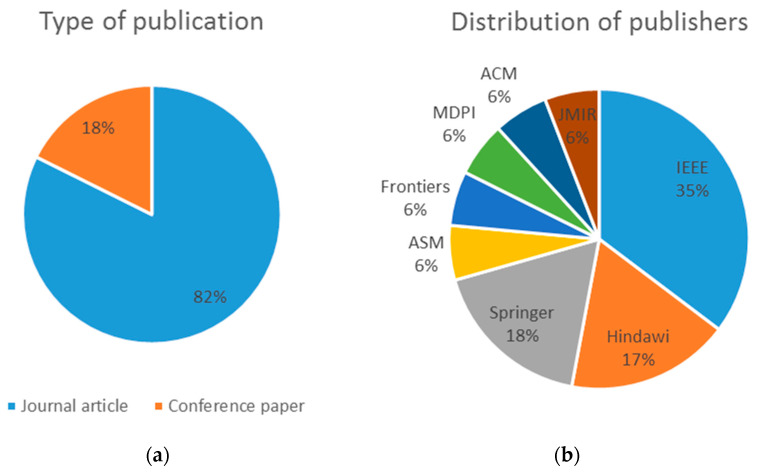
(**a**) Type of publication. (**b**) Distribution of publishers.

Table 4 shows an analysis of the bibliometric impact of the selected publications, as well as the type of publication, the authors’ affiliation and the funding received, if applicable. According to the date of publication, eight (47.1%) of the selected papers were published in 2021, six (35.3%) in 2020, two (11.8%) in 2019 and one (5.9%) in 2018 (Figure 2). Regarding citations, they have an average of 10.23 citations per paper, a minimum of zero and a maximum of 78 citations. Fourteen of the selected papers are journal articles (82.4%) and three are conference papers (17.7%) (Figure 3a).

The publisher with the greatest presence is IEEE with 29.4% of the included papers, followed by Hindawi and Springer with three (17.7%) each one; and all the others with one (5.9%) each, illustrated in Figure 3b.

**Figure 4:**
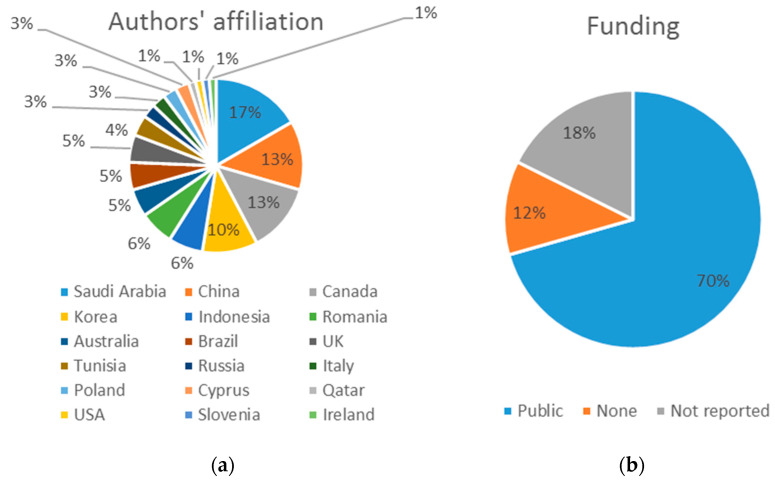
(**a**) Representation of the authors’ affiliation. (**b**) Type of funding received.

Figure 4a illustrates the affiliation of the 78 authors who signed the different papers. It shows a great diversity, with representation from 18 different countries, predominantly Saudi Arabia (17%), China (13%), Canada (13%) and Korea (10%).

Two of the 17 selected studies did not receive any type of funding (11.8%), three did not indicate whether they did or not (17.7%), and in the remaining 12 (70.6%), the authors received financial support from public sources to carry out their work (Figure 4b).

### 3.1. BC Platform Used, Access Policy and Deployment Scheme, Consensus Algorithm and End-User Delivery Support

As shown in Table 5, regarding the BC platform used, the most popular one is Hyperledger Fabric (HF) with eight works (47.1%), followed by Ethereum with five works (29.4%) and, finally, Vexanium with one work (5.9%). Five of the included studies do not report the BC platform used (29.4%). Regarding access policy and deployment scheme, nine (52.9%) of the studies use permissioned BC (i.e., only can be accessed by authorized users), of which five works (29.4%) stated that use a permissioned private BC; one work (5.9%) uses consortium BC (i.e., multiple organizations govern the P2P network hosting the BC), and three studies (17.7%) do not specify; and one study (5.9%) uses public BC (i.e., access available for all Internet users). The remaining seven works do not report the access policy and deployment scheme (41.2%). Regarding the consensus algorithm, two of the papers (11.8%) use DPoS, two use PBFT, and one paper (5.9%) uses PoW or PoA; another one (5.9%) uses PoW or DPoS; and the last one (5.9%) uses Raft Consensus. Ten of the included papers (58.8%) do not report the consensus algorithm used. Referring to the end-user delivery support, there are different options: applications through web browsers (Web App) and through smartphones (Mobile App); and decentralized applications through web browsers (Web DApps) and through smartphones (Mobile DApps). Of these options, three contributions (17.7%) use Web and Mobile App, another two ones (11.8%) use a Web App, two papers (11.8%) use Web and Mobile DApp, and another one (5.9%) uses a Web DApp and a Mobile App. Nine of the studies (52.9%) do not report the end-user delivery support.

### 3.2. Level of Use of BC Specific Properties

Regarding the specific properties of BC, nine studies (52.9%) use SC [51,53,55,56,57,60,61,62,66] and the remaining eight (47.1%) do not indicate their use. The 17 included studies justify the use of BC by supporting its special properties such as decentralization, transparency, open source, autonomy, immutability, and anonymity [8]. None of them reported the use of oracles.

### 3.3. Proposal Validation Level

Table 6 shows the proposal validation level. The level of validation of the proposal of each study has been labeled under four tags, from lowest to highest level of validation. “Theoretical proposal”, “Laboratory tests”, “Real-life testing” and “Real-life use in real situations”. “Theoretical proposal” refers to the theoretical explanation of the operation without verifying it, “Laboratory tests” refers to testing by simulating operation, without acquiring data in real situation, “Real-life testing” refers to performance testing by capturing data in a controlled laboratory environment and, “Real-life use in real situations” refers to the implementation in real situations of the developed technology. We categorized four (23.5%), eight (47.1%), two (11.8%) and two (11.8%) studies respectively under each label. Only one study (5.9%) did not describe the validation level adequately.

### 3.4. Relevant Contributions Identified

Table 7 shows the remarkable contributions to be assessed in the included studies. On the on hand, we found features belonging to the technological domain and on the other hand belonging to the physical exercise, physical activity, sport, and active ageing domain. One (5.9%) of the 17 studies makes use of cryptocurrencies as an incentive for users [51]. All the 17 studies make use of the special features of BC to provide security, immutability, and transparency of the data. Four (23.5%) of the studies use algorithms to predict future events [52,55,56,62]. Six (35.3%) of contributions capture real-time data [54,57,60,61,63,64]. And thirteen (76.5%) of papers focus on health maintenance and AA [51,53,54,55,56,57,58,60,61,62,64,65,66]. Finally, four (23.5%) of the selected works focus on sport performance [52,59,63,67]. It should be noted that no study includes NFTs to reward users, nor do they use training programs, diets, or healthy recommendations based on scientific evidence.

### 3.5. Level of Compliance with Data Protection Laws

Regarding compliance with data protection laws, only four (23.5%) of the studies explicitly report compliance. One study (5.9%) complies with HIPAA [60], two studies (11.8%) comply with GDPR [65,66], and one paper (5.9%) states that it complies with data protection laws but does not detail which one [62]. The remaining 13 studies (76.5%) do not explicitly report compliance with any of the regulating laws governing data protection.

The results of the present review may provide important implications for future research with respect to BC technology in the field of PE, PA, sport, and AA.

## 4. Discussion

In this section, we present a discussion of the data retrieved from the 17 publications analyzed. We proposed three research questions (Table 1), which are discussed here:

1. RQ1: To what extent has the use of blockchain-related solutions been explored so far in the fields of physical exercise, physical activity, sport, and active ageing?

In this systematic review, we explored publications from 1 January 2018 to 20 October 2021 in the top databases and gray literature, using the search query mentioned in Section 1. Despite performing this search in October 2021, that year contributed the largest number of publications to our final selection, as shown in Table 3, with eight publications. This may suggest that the relationship between PE, PA, sport, and active ageing with BC technology are still in their first steps of development, agreeing with previous reviews conducted in similar fields [2,3,4,5,6].

Regarding the level of development and use of BC technology, as shown in Table 3, we can see that it is at a very early stage of development as indicated by the TRL and the limited use of the special properties that BC offers.

It is, therefore, remarkable the lack of concrete application of the specific features of BC such as decentralization, transparency, autonomy, and immutability; process automation through SC; or reliability in the data included into the network through oracles [16]. In the opinion of the authors, the use of SC to automate processes and the possibility to insert data into the chain of blocks in a reliable and objective manner by oracles are differentiating elements that may play a paramount role in the near future. Another recent systematic review [7] focused on the field of healthcare and BC also tells us about the few papers found related to health education and training as well as the early stage of development of BC technology in different fields related to healthcare.

2. RQ2: From the identified use cases, what are the benefits (actual and potential) derived from this technology?

Regarding the service offered, as shown in Table 3, they range from simply providing an immutable database for data recording [59], capturing real-time data in sport to make decisions based on them [54,63], using algorithms to predict the future performance of athletes based on data stored in the BC [52] or, to predict the risk of falls [62]. Among the various advantages, we also find other works such as [51] that encourages users to lead a healthy lifestyle and meet their goals of PA through rewards in the form of cryptocurrency. In the same line is oriented the work of [65] that proposes using BC technology to capture data through questionnaires and different sensors to promote AA and assess different factors related to physical well-being. Referring to the special properties of BC, we find works ranging from the most basic, providing immutability, transparency, reliability, and security to the data; other works in which access to the data is restricted only to certain agents; to a full use to support cryptocurrencies to be used as an incentive.

Table 5 also shows the end-user delivery support referring to Web Apps, Mobile Apps, Web DApps and Mobile DApps. Only three papers use DApps. This may suggest that the full potential of this technology has not yet been fully exploited. As the reader may note, BC technology gets more interesting when DApps make the most of BC features. This way, DApps can be completely hosted in the BC P2P system and operate without the need for human intervention for decision making in the backend for business logic, since they rely on rules coded as SC [26,27]. This indicates to us, just as reported in RQ1, that this technology is in its early stages of development and its full potential has not been yet reached.

On the other hand, the inherent characteristics of BC technology also have certain drawbacks such as limited data privacy and the consequent difficulty in complying with various data protection laws. As shown in Table 8, only four of the 17 final papers included indicate that they are compliant with data protection laws in their corresponding frames. Another paper claims compliance with data protection law but does not mention which one [62]. Two of the works that claim to be compliant with data protection laws make it by storing the pointers to the data and its hash in the BC and the data payload in the distributed storage [60,62]. Another one relies on the use of firewalls, user/device authentication and communication service based on HTTPS [65] and a final one with a BC platform for consent management that allow users to monitor and manage their consent in real time and with granular variable control [66].

These gaps in data protection laws compliance may be due to the novelty of this technology. It might be appropriate to also assess the need for an update of these laws to adapt to technological advances as stated in [32]. Although the current trend is focused on the possibility of designing SC that revoke access making the data inaccessible by third parties once the data retention time has expired; the data is modified and the updated data is saved in a new block; or for any other reason [64,69,70]. Another alternative that has been discussed is the use of irreversible encryption, i.e., when the retention time of the data has expired, it can be permanently encrypted so that it cannot be reversed and retrieved. In both options, the data is not erased, it remains stored, but in an unreadable form [69].

3. RQ3: To what extent have the identified use cases taken advantage of the technical possibilities of blockchain?

Table 7 shows the noteworthy contributions of the 17 studies, in which we can see that all the papers use BC to give data security and transparency. One of the works incentivizes its users to carry out a healthy life through gamification, and cryptocurrencies. According to [71], money has a significant impact on people’s behavior. But it is remarkable the inexistence of a feature that is booming in the context of this technology such as the use of NFTs to give “medals” or “rewards” for achievements or goals of PA practice, for example. This could encourage, as in the case of [51], the practice of PA and the achievement of the proposed training goals. This lack of use of NFTs may be due to the low development of Web Apps or Mobile Apps proposed in most of the works instead of DApps. As indicated by [27], tokenization requires the implementation of DApps that enable modeling different goods in a Distributed Ledger Technology (DLT) system as digital assets that can be issued and transferred according to a predefined set of rules.

## 5. Conclusions

This systematic review aimed to identify the level of penetration of blockchain as a supporting technology in the considered domain. The promising potential of this technology led to considering it as a suitable tool in added-value services. After the research conducted, it is possible to state that the rise of BC technology is at an early stage of development and its implementation is very limited. However, some first concrete proposals for the use of this technology in this area were identified.

Most of the proposals analyzed have a very low level of technological development and provide little information about the low-level details of implementation and deployment. Also, most of them do not take advantage of specific BC features such as SC, oracles or NFTs. Scientific evidence is not used to provide training programs or healthy lifestyle recommendations. Gamification, a feature very suitable in the frame of this technology, is not leveraged to “trick” and “engage” users.

Most of the solutions reviewed are far from making the most of the potential benefits from this technology. The use of DApps, the benefits from SC, the inclusion of reliable oracles, the advanced support of gamification, or data verification possibilities are not yet fully exploited. Researchers and practitioners still have room to improve the solutions and to bring the promises of BC to actual implementations in this field. New generations of DApps are to be developed and introduced in the society to support the new and promising features of BC.

## Figures and Tables

**Table 1:** Research question aimed to answer through this systematic review.

Research Question	Statement
RQ1	To what extent has the use of blockchain-related solutions been explored so far in the fields of physical exercise, physical activity, sport, and active ageing?
RQ2	From the identified use cases, what are the benefits (actual and potential) derived from this technology?
RQ3	To what extent have the identified use cases taken advantage of the technical possibilities of blockchain?

RQ: research question.

**Table 2:** European Union TRL scale [68].

TRL	Definition
1	Basic principles observed
2	Technology concept formulated
3	Experimental proof of concept
4	Technology validated in lab
5	Technology validated in relevant environment (industrially relevant environment in the case of key enabling technologies)
6	Technology demonstrated in relevant environment (industrially relevant environment in the case of key enabling technologies)
7	System prototype demonstration in operational environment
8	System complete and qualified
9	Actual system proven in operational environment (competitive manufacturing in the case of key enabling technologies; or in space)

**Table 3:** Synthesis of the studies. Ordered in chronological order and according to scope.

Paper	Scope	Service Proposal	TRL	Information Stored	Use of BC Properties
Alsalamah et al. (2021) [51]	Fitness and healthcare	Incentivizing PA practice by rewarding cryptocurrencies.	TRL-3	Actual data.	Cryptocurrencies support.
Frikha et al. (2021) [53]	Fitness and healthcare	Diagnosis and treatment of various diseases easily and cost-effectively through data stored in eHR.	TRL-4	Actual data.	Authorized accessibility to health and fitness data for the different agents involved.
Jamil, Qayyum et al. (2021) [56]	Fitness and healthcare	Suggesting training and diet programs according to the subject’s body parameters. Also support data transfer among sport centers.	TRL-4	Actual data.	Data accountability, improve data privacy and accessibility.
Jamil, Kahng et al. (2021) [55]	Fitness and healthcare	Suggesting training and diet programs according to biomedical parameters.	TRL-3	Actual data.	Data accountability, improve data privacy and accessibility.
Yu (2021) [67]	Sport	Improved and guided training sessions through wrist sensors physiological data about athletes’ fitness status.	TRL-3	Actual data.	Transparency and integrity of the user’s personal data.
Cao et al. (2021) [52]	Sport	Performance predictions and better training programs.	TRL-4	Actual data.	Immutability of data about training and ensuring accessibility at any time.
Ma (2021) [58]	Sport	Support to analyze data on users walking patterns.	TRL-4	Actual data.	Sharing, storing, and protecting the user’s personal privacy data.
Mulyati et al. (2020) [59]	Sport	Transparency and reliability to the assessments made by judges in Taekwondo belt promotions.	TRL-6	Actual data.	Transparency, reliability, and immutability to prevent loss or manipulation of data.
Shan & Mai (2020) [63]	Sport	Real time detection and management of athlete’s fitness status.	TRL-4	Actual data.	Privacy of data collection and transmission.
Hong et al. (2020) [54]	Sport	Support tactical decisions in competitions.	TRL-2	Actual data.	Authorized accessibility to player data.
Spinsante et al. (2021) [65]	Active ageing	AA promotion, to make the elderly more independent and to evaluate their level of PA and quality of life through their App.	TRL-4-5	Actual data.	Management of permissions and control of access to the collection of health information and the use of personal data.
Khezr et al. (2020) [57]	Active ageing	Detecting anomalies on users’ behavior when their normal daily life patterns are non-conformant.	TRL-4	Actual data.	Data protection, maintenance, sharing and accessibility to authorized agents.
Velmovitsky et al. (2020) [66]	Active ageing	Control over informed consent for active assisted living.	TRL-2	Actual data.	Immutability, time stamped, transparency and control over user data.
Rahman et al. (2020) [61]	Active ageing	Control over intelligent elements of the house by means of intelligent gesture detection devices.	TRL-4	Pointers and hash of actual data.	Security, trust, digital identity, privacy preservation and accessibility to raw multimedia and gesture data
Silva et al. (2019) [64]	Active ageing	Control over data management and user access.	TRL-4	Actual data.	Privacy and authorized accessibility to data.
Rupasinghe et al. (2019) [62]	Active ageing	Identify risk factors for falls and a prediction model using SC.	TRL-3	Pointers and hash of actual data.	Immutability, transparency, and authorized accessibility to data.
Rahman et al. (2018) [60]	Active ageing	Suggest therapies according to the user’s treatment needs.	TRL-4	Pointers and hash of actual data.	Privacy, ownership, generation, storage, and sharing therapeutic data.

**Table 4:** Bibliometric overview of selected studies.

Reference	Year	Citation Counter	Publication Type	Publisher	Authors’ Affiliation	Funding
Alsalamah et al. [51]	2021	0	Journal article	Frontiers.	Saudi Arabia	Public
Cao et al. [52]	2021	0	Journal article	IEEE	China	Public
Frikha et al. [53]	2021	6	Journal article	Hindawi	Tunisia and Saudi Arabia	Public
Hong et al. [54]	2020	0	Journal article	ASM	Korea	NR
Jamil et al. [55]	2021	10	Journal article	MDPI	Korea	Public
Jamil et al. [56]	2021	4	Journal article	Tech Science Press	Korea, Saudi Arabia, Pakistan, and Russia	None
Khezr et al. [57]	2020	1	Conference Paper	IEEE	Canada	NR
Ma [58]	2021	1	Journal article	Springer	China	Public
Mulyati et al. [59]	2020	9	Conference Paper	IEEE	Indonesia	Public
Rahman et al. [61]	2020	3	Conference Paper	IEEE	Canada, UK, and Qatar	Public
Rahman et al. [60]	2018	78	Journal article	IEEE	Saudi Arabia, USA, and UK	Public
Rupasinghe et al. [62]	2019	20	Journal article	ACM	Australia	Public
Shan & Mai [63]	2020	4	Journal article	Springer	China	None
Silva et al. [64]	2019	27	Journal article	Hindawi	Brazil	Public
Spinsante et al. [65]	2021	2	Journal article	Springer	Italy, Poland, Romania, and Cyprus	Public
Velmovitsky et al. [66]	2020	8	Journal article	JMIR Publications	Canada	Public
Yu [67]	2021	2	Journal article	Hindawi	China	NR

NR: Not reported.

**Table 5:** Characteristics of the BC networks used in the selected studies.

Paper	Platform	Access Policy and Deployment Scheme	Consensus Algorithm	End-User Delivery Support
Alsalamah et al. (2021) [51]	Ethereum	Permissioned private	NR	Web DApp and Mobile App
Cao et al. (2021) [52]	NR	NR	NR	NR
Frikha et al. (2021) [53]	Ethereum	Permissioned private	PoW and PoA	Web and Mobile App
Hong et al. (2020) [54]	Hyperledger Fabric	Permissioned	PBFT	NR
Jamil et al. (2021a) [55]	Hyperledger Fabric	Permissioned	PBFT	Web App
Jamil et al. (2021b) [56]	Hyperledger Fabric	Permissioned	NR	Web App
Khezr et al. (2020) [57]	Hyperledger Fabric	NR	NR	NR
Ma (2021) [58]	NR	NR	DPoS	NR
Mulyati et al. (2020) [59]	Vexanium	Public	DPoS	Web and Mobile DApp
Rahman et al. (2018) [60]	Ethereum and Hyperledger Fabric	Permissioned private	PoW and DPoS	NR
Rahman et al. (2020) [61]	Ethereum and Hyperledger Fabric	Permissioned private	NR	Web and Mobile DApps
Rupasinghe et al. (2019) [62]	NR	Permissioned consortium	NR	NR
Shan & Mai (2020) [63]	NR	NR	NR	NR
Silva et al. (2019) [64]	Ethereum	NR	NR	Web and Mobile App
Spinsante et al. (2021) [65]	Hyperledger Fabric	NR	NR	Web and Mobile App
Velmovitsky et al. (2020) [66]	Hyperledger Fabric	Permissioned private	Raft consensus	NR
Yu (2021) [67]	NR	NR	NR	NR

NR: Not reported.

**Table 6:** Validation level.

Validation Level	References
Theoretical proposal	[54,55,62,66]
Laboratory tests	[51,52,53,56,57,58,60,61]
Real-life testing	[63,64]
Real-life use in real situations	[59,65]
Not described	[67]

**Table 7:** Relevant contributions.

	Technological Domain					Physical Exercise, Physical Activity, Sport, and Active Ageing Domain		
	Incentivize with Crypto Currencies	Use NFT	Provide Security and Transparency with the Use of BC	Use Predictive Algorithms	Real-Time Data	Related to Healthy Living, Healthcare and Ageing	Evidence-Based Training Programs	Sport Performance
Alsalamah et al. (2021) [51]	✓	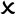	✓	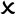	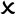	✓	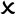	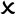
Cao et al. (2021) [52]	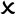	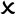	✓	✓	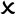	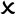	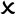	✓
Frikha et al. (2021) [53]	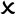	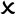	✓	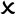	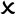	✓	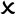	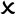
Hong et al. (2020) [54]	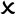	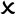	✓	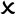	✓	✓	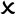	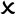
Jamil et al. (2021a) [55]	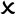	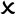	✓	✓	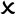	✓	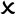	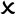
Jamil et al. (2021b) [56]	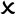	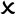	✓	✓	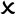	✓	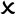	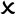
Khezr et al. (2020) [57]	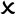	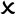	✓	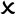	✓	✓	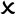	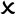
Ma (2021) [58]	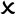	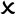	✓	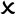	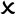	✓	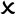	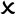
Mulyati et al. (2020) [59]	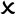	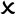	✓	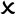	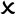	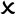	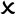	✓
Rahman et al. (2018) [60]	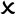	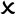	✓	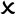	✓	✓	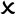	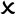
Rahman et al. (2020) [61]	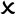	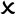	✓	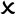	✓	✓	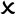	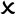
Rupasinghe et al. (2019) [62]	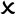	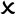	✓	✓	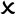	✓	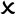	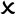
Shan & Mai (2020) [63]	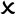	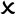	✓	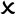	✓	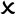	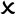	✓
Silva et al. (2019) [64]	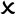	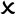	✓	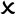	✓	✓	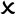	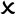
Spinsante et al. (2021) [65]	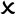	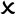	✓	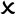	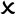	✓	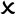	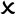
Velmovitsky et al. (2020) [66]	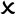	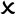	✓	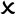	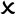	✓	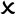	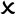
Yu (2021) [67]	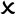	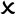	✓	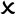	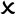	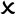	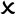	✓

✓: The paper meets the condition; 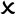: The paper does not meet the condition.

**Table 8:** Compliance with data protection laws.

Compliance with Data Protection Laws	References
Yes	[60,62,65,66]
Not reported	[51,52,53,54,55,56,57,58,59,61,63,64,67]

## Data Availability

Not applicable.
